# Integrating UpToDate with case-based learning enhances residents’ clinical decision-making and training satisfaction in critical care training

**DOI:** 10.3389/fmed.2026.1743395

**Published:** 2026-01-16

**Authors:** Daowei Zhang, Wenbin Sun, Xinxing Lu

**Affiliations:** Department of Critical Care Medicine, The Affiliated Taizhou People's Hospital of Nanjing Medical University, Taizhou, Jiangsu, China

**Keywords:** case based lerning, CDS tools, clinical decision-making, critcal care education, UpToDate

## Abstract

**Objective:**

Case-based learning (CBL) enhances medical students’ clinical competence but faces limitations like reliance on outdated information and insufficient evidence-based decision-making support. UpToDate, an evidence-based, continuously updated clinical resource, provides current data, standardized pathways, and improved application skills. Integrating UpToDate into CBL offers potential for enhancing the accuracy, timeliness, and multidisciplinary nature of learning. This study developed the “UpToDate-CBL” model for critical care education and evaluated its effectiveness in clinical teaching.

**Methods:**

Sixty standardized residency trainees were randomized to a CBL group (*n* = 30) or an UpToDate-CBL group (*n* = 30). The effectiveness of the UpToDate-CBL model in comparison with CBL model by objectively measuring the students’ theoretical knowledge and clinical skills. Additionally, the quality of teaching was evaluated subjectively through anonymous questionnaires completed by the students.

**Results:**

No significant differences existed in theoretical knowledge (86.67 ± 5.02 vs. 84.53 ± 3.79, *p* = 0.097) or clinical skills scores (84.40 ± 4.83 vs. 83.47 ± 4.52, *p* = 0.442) between groups. However, the UpToDate-CBL group showed significantly higher scores in clinical decision-making (43.47 ± 3.67 vs. 41.20 ± 2.55, *p* = 0.011) and clinical parameter interpretation (44.87 ± 3.95 vs. 42.27 ± 3.78, *p* = 0.012). Moreover, self-assessment scores in the UpToDate-CBL group for learning interest (3.83 ± 0.65 vs. 3.20 ± 0.71, *p* = 0.001), self-learning ability (3.27 ± 0.74 vs. 2.83 ± 0.75, *p* = 0.034), problem-solving ability (3.50 ± 0.86 vs. 2.87 ± 0.73, *p* = 0.004), and practical ability (3.57 ± 0.82 vs. 2.93 ± 0.69, *p* = 0.003) were all significantly higher than in the CBL group. Teaching satisfaction was also significantly higher in the UpToDate-CBL group (90.00% vs. 70.0%, *p* < 0.05).

**Conclusion:**

Compared with the CBL model, the UpToDate-CBL approach significantly enhances students’ proficiency in interpreting clinical parameters and clinical decision making, while also effectively fostering their motivation for self-directed learning. This approach demonstrates distinct advantages in clinical reasoning training within the context of critical care medicine; nevertheless, it cannot achieve breakthroughs in teaching basic knowledge and clinical skills.

## 1 Introduction

Critical care medicine, characterized by its rapid evolution and high-stakes nature, demands that clinicians master foundational theoretical knowledge, advanced life support techniques, and effective multidisciplinary collaboration (1). Consequently, training within this discipline must cultivate complex clinical thinking, and the ability to make time-sensitive decisions under pressure. However, conventional teaching models struggle to meet such requirements.

Case-based learning (CBL) effectively meets educational needs by grounding instruction in real clinical scenarios. Through a focus on learner-centered case analysis, structured group discussions, and facilitator support, CBL significantly improves trainees’ abilities to analyze complex critical illnesses and develop sound clinical reasoning for diagnosis and treatment (2). In contrast, traditional lecture-based learning—though effective for systematic knowledge delivery—struggles to mirror the dynamic pressures and intricate decision-making in real-world critical care (3). Integrating evidence-based clinical decision support tools (CDS) like UpToDate into clinical education has emerged as a promising trend. These digital resources offer key pedagogical benefits: real-time access to current guidelines, bridging experiential knowledge gaps, and fostering adaptable frameworks (4). Additionally, their standardized pathways provide educators with objective benchmarks to evaluate trainees’ reasoning in dynamic simulations, reducing subjectivity from variable preceptor experience (5). This study integrates the UpToDate knowledge base into critical care CBL. Using quantitative metrics, we systematically examine the practical benefits of augmenting CBL with CDS in critical care training.

## 2 Methods

### 2.1 Study setting

This educational randomized controlled trial was conducted within the Department of Critical Care Medicine at Taizhou People’s Hospital Affiliated to Nanjing Medical University and has been approved by the Ethics Committee of Taizhou People’s Hospital at 06/10/2023 (no. JX20230610). All teaching activities were conducted by the same senior associate chief physician.

### 2.2 Participants

**Figure 1:**
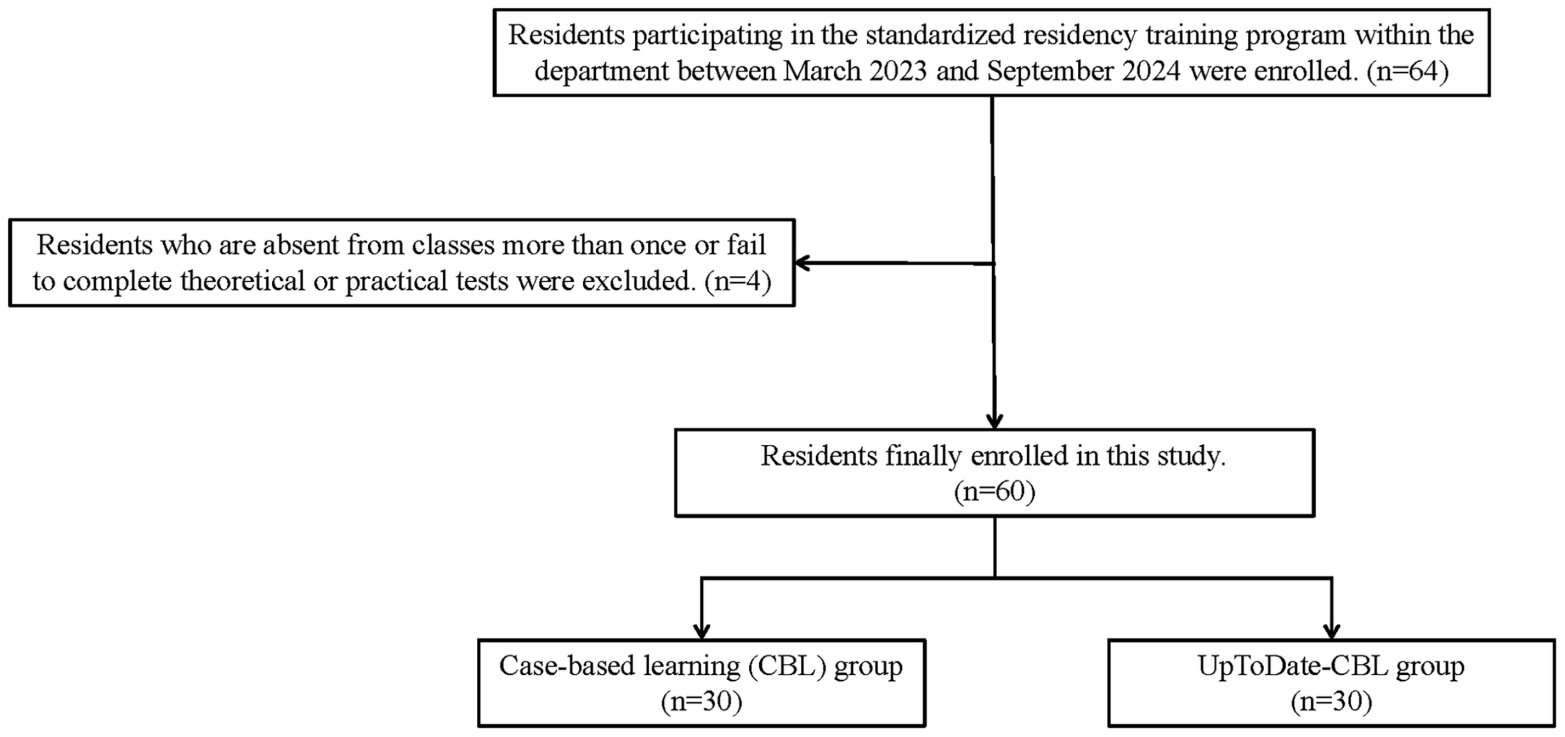
Flowchart of the subject selection.

Sample size was calculated *a priori* to detect a mean difference of 3 points in clinical decision-making scores (SD = 4) with *α* = 0.05 and power = 0.80, yielding 28 participants per group (6); 30 were enrolled to allow for 5% attrition. A total of 64 residents participating in the standardized residency training program within the department were enrolled in the study from 07/03/2023 to 09/27/2024. Residents who are absent from classes more than once or fail to complete theoretical or practical tests were excluded from the study (*n* = 4, Figure 1). The cohort comprised 38 males and 22 females, aged 24 to 29 years (mean 25.63 ± 1.56 years), with clinical experience ranging from 1 to 7 years (mean 2.7 years). All participants had a primary background in internal medicine and had completed systematic undergraduate medical education, and all provided informed consent. Participants were randomly assigned to either the CBL group (*n* = 30, 20 males, 10 females) receiving traditional CBL instruction or the UpToDate-CBL group (*n* = 30, 18 males, 12 females) receiving instruction integrating UpToDate with CBL using the random number table method. Allocation concealment was implemented using sequentially numbered, opaque, sealed envelopes. After baseline assessments, participants were assigned a computer-generated random ID. The randomization sequence was kept concealed by teaching secretary. Upon enrollment, each participant received the next sealed envelope, which contained their group assignment.

### 2.3 Teaching interventions

#### 2.3.1 CBL group

Three days before each session the instructor provided students with foundational theoretical materials and a representative case study accompanied by critical thinking questions. Students independently prepared using textbooks and literature to formulate case analyses. During the 2 h class session, students collectively reviewed the medical history, physical findings, and bedside imaging from preselected cases under instructor facilitation. They then engaged in intensive subgroup discussions (6 students per group) to develop diagnoses, differentials, and management strategies, with the instructor offering only procedural guidance rather than direct answers. Each subgroup subsequently presented their analysis through two primary presenters, while other members supplemented key points. The instructor concluded by evaluating presentations, resolving queries, and synthesizing core theoretical principles.

#### 2.3.2 UpToDate-CBL group

For this enhanced model, the instructor distributed a digital learning package 3 days prior containing core theoretical frameworks, complete clinical case data, and UpToDate Clinical Pathways documents. Students were specifically instructed to identify at least two significant discrepancies between UpToDate recommendations and textbook content. The session commenced with a 20-min instructor-led demonstration of mobile-based UpToDate navigation tailored to the case. Subsequently, over 45 min, students merged conventional case discussion with real-time evidence application: They utilized UpToDate’s DDx tool to compare epidemiological profiles during differential development, and conducted evidence-based debates on contentious management points by citing guideline entries and recent studies in assigned roles. Each group then delivered a 15-min structured presentation focused on clinical reasoning. Throughout this process, the instructor monitored evidence implementation quality in real-time, concluding the session by addressing evidence-application pitfalls while demonstrating advanced UpToDate features.

### 2.4 Outcome assessment

#### 2.4.1 Objective evaluation

(1) Fundamental knowledge test (35%): A 100-question, closed-book examination administered via the Jiangsu Province Standardized Residency Training Online Examination System. Topics covered core critical care concepts (Max Score: 100). (2) Clinical skills assessment (35%): Performance evaluation of essential critical care procedures, including endotracheal intubation, central venous catheter insertion, and arterial line insertion (Max Score: 100). (3) Clinical data interpretation (15%): Assesses the ability to interpret clinical parameters relevant to critical care scenarios (Max Score: 50). (4) Clinical decision-making (15%): Evaluates the ability to formulate appropriate diagnostic and therapeutic plans in critical care contexts (Max Score: 50). All outcome assessments were scored by two independent assessors blinded to group allocation. If the scores given by the two raters differed substantially, another senior member of the teaching team would conduct a re-evaluation. Participants were instructed not to disclose group allocation to assessors.

#### 2.4.2 Subjective evaluation

Participants completed an anonymous questionnaire assessing self-perceived competency gains and teaching satisfaction. Self-perceived gains (in interest in learning, self-directed learning ability, problem-solving skills, clinical skills, and overall competency) were rated on a 5-point Likert scale (7) (1 = Strongly Disagree, 2 = Disagree, 3 = Neutral, 4 = Agree, 5 = Strongly Agree).

Teaching satisfaction was categorized as Satisfied, Generally Satisfied, or Dissatisfied. The Overall Satisfaction Rate was calculated as [(Number of Satisfied + Number of Generally Satisfied)/Total Participants] × 100%.

### 2.5 Statistical analysis

Data analysis was performed using SPSS Statistics software 27.0. Continuous data are presented as mean ± standard deviation. Normality of distribution was assessed using Kolmogorov–Smirnov tests. Independent samples t-tests were used for inter-group comparisons; Mann–Whitney U tests were used for non-normally distributed data. The correction method uses Welch’s test. Teaching satisfaction was expressed as a percentage (%). Baseline comparisons employed independent *t*-tests. All tests were two-tailed, with a *p*-value < 0.05 considered statistically significant.

## 3 Results

### 3.1 Comparison of basal knowledge assessment results between two groups

Baseline characteristics, including age, gender, years in practice and academic qualification, showed no statistically significant differences between the groups (Table 1). Compared with the CBL group, the UpToDate-CBL group achieved significantly higher scores in clinical decision-making (43.47 ± 3.67 vs. 41.20 ± 2.55, *p* = 0.007), clinical parameter interpretation (44.87 ± 3.95 vs. 42.27 ± 3.78, *p* = 0.012), and total assessment (86.37 ± 5.06 vs. 83.84 ± 3.84, *p* = 0.033), whereas no statistically significant differences were observed in theoretical knowledge (86.67 ± 5.02 vs. 84.53 ± 3.79, *p* = 0.0972) or clinical skills (84.40 ± 4.83 vs. 83.47 ± 4.52, *p* = 0.442). Table 2 presents the detailed data. This evidence demonstrates that integrating UpToDate with CBL significantly enhances clinical parameter interpretation and decision-making competencies, while providing no marked improvement in theoretical knowledge or clinical skills compared to standard CBL methodology.

**Table 1:** Baseline characteristics of participants.

Characteristics	CBL group (*n* = 30)	UpToDate-CBL group (*n* = 30)	*p* value
Gender, *n* (%)			0.789
Male	20 (66.67)	18 (60%)	
Female	10 (33.33)	12 (40%)	
Age	25.47 ± 1.55	25.73 ± 1.57	0.473
Academic qualification, *n* (%)			0.774
Bachelor’s	22 (73.33)	21 (70.0)	
Master’s	8 (26.67)	9 (30.0)	
Years in practice	2.63 ± 1.59	2.86 ± 1.61	0.520
CDS usage history	0	0	
AI usage history	10	12	0.789

Normality of distribution was assessed using Kolmogorov–Smirnov tests. Independent samples *t*-test or Mann–Whitney U test was used for inter-group comparisons. Quantitative data are represented as mean ± SD. *p* < 0.05 indicates significant difference.

**Table 2:** Comparison of students’ assessment results.

Elements	CBL group (*n* = 30)	UpToDate-CBL group (*n* = 30)	Cohen’s *d*	95% CI	*p* value
Overall scores	83.84 ± 3.84	86.37 ± 5.06	−0.56	0.2077–4.859	**0.0330**
Theoretical knowledge	84.53 ± 3.79	86.67 ± 5.02	−0.48	−0.1679-4.435	0.0972
Clinical skills	83.47 ± 4.52	84.40 ± 4.83	−0.20	−1.482-3.349	0.4424
Clinical judgment	41.20 ± 2.55	43.47 ± 3.67	−0.72	0.6276–3.906	**0.0109**
Clinical parameter profiling	42.27 ± 3.78	44.87 ± 3.95	−0.67	0.6013–4.599	**0.0117**

Normality of distribution was assessed using Kolmogorov–Smirnov tests. Independent samples *t*-test or Mann–Whitney U test was used for inter-group comparisons. Quantitative data are represented as mean ± SD. *p* < 0.05 indicates significant difference. Bold font indicates statistical significance.

### 3.2 Student self-assessment

All 60 administered teaching quality questionnaires were returned and deemed valid. Students in the UpToDate-CBL group reported significantly higher self-assessment scores than the CBL group across all domains: learning interest (3.83 ± 0.65 vs. 3.20 ± 0.71, *p* = 0.0014), self-directed learning ability (3.27 ± 0.74 vs. 2.83 ± 0.75, *p* = 0.0338), problem-solving capacity (3.50 ± 0.86 vs. 2.87 ± 0.73, *p* = 0.0044), practical ability (3.57 ± 0.82 vs. 2.93 ± 0.69, *p* = 0.0026), and comprehensive competency (14.17 ± 2.59 vs. 11.83 ± 2.64, *p* = 0.0007). Table 3 presents the detailed data. Complete results are shown in Table 2. These outcomes suggest that the UpToDate-CBL approach significantly enhances students’ intrinsic learning motivation and self-perceived skill development across multiple dimensions compared to conventional CBL instruction.

**Table 3:** Comparison of self-evaluation between two group students.

Self-evaluation	CBL group (*n* = 30)	UpToDate-CBL group (*n* = 30)	Cohen’s *d*	95% CI	*p* value
Study interest	3.20 ± 0.71	3.83 ± 0.65	−0.93	0.2808–0.9858	**0.0014**
Self-learning ability	2.83 ± 0.75	3.27 ± 0.74	−0.58	0.04923–0.8174	**0.0338**
Problem solving ability	2.87 ± 0.73	3.50 ± 0.86	−0.79	0.2205–1.046	**0.0044**
Clinical Competence	2.93 ± 0.69	3.57 ± 0.82	−0.84	0.2419–1.025	**0.0026**
Comprehensive quality	11.83 ± 2.64	14.17 ± 2.59	−0.92	1.027–3.639	**0.0007**

Normality of distribution was assessed using Kolmogorov–Smirnov tests. Independent samples t-test or Mann–Whitney U test was used for inter-group comparisons. Quantitative data are represented as mean ± SD. *p* < 0.05 indicates significant difference. Bold font indicates statistical significance.

### 3.3 Teaching satisfaction

Satisfaction survey results revealed significantly higher overall satisfaction in the UpToDate-CBL group (90.0%: 60.0% Satisfied + 30.0% Generally Satisfied) compared to the CBL group (70.0%: 36.7% Satisfied + 33.3% Generally Satisfied) (*p* = 0.036, Table 4). These data demonstrate superior acceptance and perceived educational value of the UpToDate-CBL methodology among learners.

**Table 4:** Comparison of course satisfaction levels between the two student groups.

Groups	Satisfied	Basically satisfied	Unsatisfied	Satisfaction rate	*Z* score	*p* value
CBL	11 (36.7%)	10 (33.3%)	9 (30.0%)	70.0%	**−2.096**	**0.036**
UpToDate-CBL	18 (60%)	9 (30%)	3 (10%)	90%		

Independent sample Mann–Whitney U test was used for inter-group comparisons. *p* < 0.05 indicates significant difference. Bold font indicates statistical significance.

## 4 Discussion

This study addresses critical challenges in traditional ICU medical education by developing and evaluating an integrated CBL and UpToDate clinical decision support teaching model for standardized residents. Our results demonstrate that the UpToDate-CBL integration significantly enhances residents’ abilities in clinical data interpretation and evidence-based decision-making. Additionally, the model significantly enhances motivation for self-directed learning and results in higher teaching satisfaction compared to conventional CBL method, confirming its effectiveness as a valuable strategy for training in critical care.

Critical care medicine poses a significant challenge in clinical medical education due to its interdisciplinary nature and the urgent demands of managing critically ill patients. This field requires the integration of complex pathophysiological mechanisms, the interpretation of dynamic monitoring data, and the development of real-time, evidence-based decision-making skills to address patient needs for precise life support and tailored intervention strategies (8). Critical care training systems in China are structured in multiple tiers, which include undergraduate general education, standardized residency training, and specialized advanced fellowship programs (9). Standardized residents, when compared to undergraduates, have a more systematic understanding of critical care pathophysiology due to their foundational clinical rotations. However, there are still significant gaps in their knowledge compared to specialists, particularly in mastering advanced skills such as selecting appropriate mechanical ventilation strategies for acute respiratory failure and determining eligibility for extracorporeal life support. Therefore, it is crucial to enhance the logical rigor and precision of clinical decision-making among residents to support their professional development. This need for improvement is a key reason why we chose this cohort as our study population.

Currently, LBL remains the mainstream in teaching, especially in developing countries, which has limitations in effectively evaluating trainees’ abilities to respond to clinical emergencies or enhance teaching quality (10). This teacher-centric, one-way method fails to replicate the collaborative dynamics of a real-world ICU, thereby hindering the development of trainees’ self-directed learning and critical clinical thinking skills (11). Additionally, the high-pressure environment necessary for effective clinical decision-making does not align with the passive nature of traditional instruction; trainees frequently miss opportunities for independent decision-making, such as initiating orders or adjusting ventilator settings during actual resuscitation scenarios (12). CBL, or Case-Based Learning, is a teaching method that uses real clinical cases to enhance students’ clinical reasoning and problem-solving skills (13). In the context of critical care education, CBL not only sparks student interest but also fosters teamwork and communication abilities (14). However, this approach has its challenges, such as the significant time required for case preparation, the variability in content quality that can arise from differing instructor expertise, and the difficulties students may encounter when dealing with complex cases. Additionally, differences in students’ pre-class material review abilities lead to significant variation in the knowledge they acquire. This results in numerous ineffective discussions during the CBL discussion phase—even causing deviations from originally set objectives and wasting considerable time. At the same time, it places higher demands on instructors, requiring them to continuously guide discussions back to standard diagnostic and therapeutic strategies.

The informatization and digitalization of healthcare have emerged as core drivers of global advancement, necessitating a paradigm shift in medical education to keep pace with this trend. Early explorations have already highlighted the value of digital learning: a 2014 U.S. survey of emergency medicine residents revealed that medical podcasts were perceived as the most effective learning modality compared to textbooks, journals, or search engines, preliminarily validating the potential of informatized education (15). With the proliferation of mobile technology, a 2017 study further demonstrated that 76.7% of respondents actively used medical apps—including Epocrates, UpToDate, and UWorld—during their learning, reporting that clinically focused tools and self-directed case-based learning were more effective for knowledge acquisition (16). A 2023 study at Rennes University Hospital, which developed the multi-platform app “DansMaBlouse” for anesthesia and critical care residents, showed that applications integrating personal and shared resources could deliver precise knowledge support at the point of care, significantly enhancing learning efficiency and clinical decision-making (17). A 2025 review of dental education further emphasized the transformative potential of smartphone apps in endodontic education, enabling flexible learning and skill reinforcement, though challenges such as variable quality and limited validation remain to be addressed (18).

Recent advances in artificial intelligence (AI) have injected new momentum into medical education. A 2024 review by Schubert underscored that AI-driven tools can revolutionize educational models through personalized tutoring (19); similarly, Somerville’s work on nursing education confirmed the immense potential of AI in transforming nursing practice and pedagogy (20). However, the application of AI in cultivating clinical decision-making skills among medical students remains contentious: a key limitation lies in the inability to verify whether AI recommendations are grounded in authentic research evidence or authoritative clinical guidelines—an area where CDS tools, exemplified by UpToDate, hold distinct advantages. By structurally integrating evidence-based medicine and guidelines, CDS provides a transparent and reliable knowledge anchor for medical education. This advantage is particularly pronounced in emergency and critical care settings: a study in emergency departments demonstrated that CDS effectively facilitates quality improvement in primary palliative care (21), while a survey at the University of Ottawa further corroborated that medical students and residents widely regard trustworthy clinical resource apps like UpToDate as pivotal for professional growth, with the highest reported usage frequency (22). Collectively, in the current landscape where AI technology is still maturing, CDS tools, with their evidence-based framework and clinical adaptability, remain an irreplaceable support system in medical education.

UpToDate, a leading global resource for clinical decision support, offers access to the latest and most reliable evidence-based medical information (23). By integrating UpToDate with CBL, many of these limitations can be effectively mitigated. In this combined teaching model, instructors can draw from UpToDate’s extensive library of high-quality case vignettes and meticulously synthesized evidence to create more scientifically sound and standardized case scenarios, ensuring that the content is both accurate and current (5). During discussions of these cases, students have the opportunity to access UpToDate in real-time, allowing them to find best practices and the most recent research related to the conditions being studied, which enhances their clinical decision-making skills and accelerates their learning (24). Notably, the use of UpToDate helps level out students’ background knowledge before and during class, making the entire course more compact and coherent. More importantly, based on the cutting-edge background knowledge acquired, students’ discussion depth is further enhanced—they can raise more unresolved scientific questions from existing clinical issues, sparking enthusiasm for independent exploration after class. This conclusion aligns with the evaluation results of students’ self-learning ability. Through the integration of CBL and UpToDate, students not only engage in case analysis to develop their clinical reasoning but also utilize UpToDate to incorporate the latest medical evidence, leading to a more profound connection between theoretical knowledge and practical application.

## 5 Limitations

This study has several limitations that should be acknowledged. First, incorporating UpToDate into CBL significantly increases the demands on instructors to effectively manage multidisciplinary teaching, since the complexity of critical care scenarios makes it difficult to maintain strict control over educational variables. Second, while UpToDate offers immediate access to guidelines, it may unintentionally encourage trainees to become overly dependent on it, which could impede their understanding of the fundamental pathophysiology that informs clinical decisions. Third, the assessment primarily concentrated on short-term learning outcomes, and the interference of the novelty effect cannot be ruled out, leaving questions about the long-term retention of knowledge and its impact on sustained clinical competency. Finally, the use of CDS tools such as UpToDate incurs certain costs, thus posing practical difficulties for their promotion in general hospitals in developing countries. Future research should investigate the creation of integrated digital teaching platforms to improve implementation, enhance learning outcomes, and specifically tackle the issue of over-reliance, while also including longer-term follow-up assessments.

## 6 Conclusion

This study introduces an UpToDate-CBL integrated teaching model for critical care education, demonstrating preliminary benefits in short-term outcomes: improved clinical parameter interpretation, decision-making, learning motivation, problem-solving, and self-directed skills, alongside higher teaching satisfaction versus standalone CBL. While the model shows promise as an evidence-based approach, its implementation faces challenges—including increased instructor demands for multidisciplinary management, potential trainee over-reliance on UpToDate, and cost-related barriers to adoption in resource-limited settings. Notably, the assessment focused on short-term effects, with unaddressed novelty effects and uncertain long-term knowledge competency impacts. Future work should prioritize integrated digital platforms to mitigate over-reliance, optimize implementation, and incorporate long-term follow-up to validate sustained benefits. Thus, the UpToDate-CBL model warrants cautious adoption and further investigation in diverse clinical training contexts.

## Data Availability

The raw data supporting the conclusions of this article will be made available by the authors, without undue reservation.
